# Influence of rigidity of retainers on dynamic behavior of implant-supported removable partial dentures

**DOI:** 10.1186/s40729-020-00260-4

**Published:** 2020-10-22

**Authors:** Toshifumi Nogawa, Masayasu Saito, Naomichi Murashima, Yoshiyuki Takayama, Atsuro Yokoyama

**Affiliations:** 1 Preventive Dentistry, Hokkaido University Hospital, Sapporo, Japan; 2 Oral Functional Prosthodontics, Division of Oral Functional Science, Facility of Dental Medicine, Hokkaido University, Sapporo, Japan; 3 Oral Functional Prosthodontics, Division of Oral Functional Science, Graduate School of Dental Medicine, Hokkaido University, Sapporo, Japan; 4 Oral Rehabilitation, Hokkaido University Hospital, Sapporo, Japan

**Keywords:** Implant-supported partial dentures, Removable partial dentures, Direct retainers, Dynamic behavior, Rigidity of connection

## Abstract

**Background:**

Implant-supported removable partial dentures (ISRPDs) are an effective treatment for partially edentulous patients. ISRPDs improve patients’ satisfaction and oral function to a greater extent than RPDs by improving denture stability and enhancing support. However, the effect of a type of direct retainer on displacement of the abutment teeth and dentures in ISRPDs remains unclear. Therefore, we made a resin mandibular model of unilateral mandibular distal-extension partial edentulism for mechanical simulation and compared the dynamic behavior of the abutment teeth and the denture base among different tooth-borne retainers with various rigidities for RPDs and ISRPDs.

**Methods:**

A resin mandibular model for mechanical simulation that had unilateral mandibular distal-extension edentulism and was missing the first molar, second molar, first premolar, and second premolar, and a denture fabricated from the patient’s computed tomography images were used. Three types of direct retainers with different connecting rigidities were evaluated. The vertical displacement of the denture base and buccal and lingual sides and the mesial displacement of the abutment teeth were measured.

**Results:**

Regardless of the rigidity of the direct retainers and loading positions, the displacement of the denture bases in the ISRPDs was significantly smaller than that in the RPDs (*P* < 0.001). There was no significant difference in vertical displacement of the denture bases among direct retainers with various connecting rigidities in the ISRPDs. Conversely, horizontal displacement of the abutment teeth in both the RPDs and ISRPDs tended to be larger with the cone crown telescope, which has high rigidity, than with the cast cingulum rest and wire clasp, which have much lower rigidities.

**Conclusion:**

Our results suggested that cast cingulum rest and wire clasps as direct retainers are appropriate ISRPDs to minimize denture movement and suppress displacement of the remaining teeth in patients with unilateral mandibular distal-extension partial edentulism.

## Background

The prosthetic methods for partial edentulism generally include fixed partial dentures, removable partial dentures (RPDs), and implant-supported fixed prostheses (ISFPs). For patients who are missing many teeth, RPDs or ISFPs are inserted into the partial edentulous space. However, the teeth adjacent to the partial edentulous space in which an RPD has been inserted have a reported risk of tooth loss because of occlusal overloading [1]. Conversely, ISFPs do not overload the remaining teeth. However, if the partial edentulous space is wider or absorption of the residual ridge is severe, the cost of implant treatments might be higher because of the need for many implants, or inserting implants might be difficult because of the lack of bone volume.

Implant-supported RPDs (ISRPDs) are an effective treatment for partially edentulous patients. ISRPDs improve patients’ oral health-related quality of life, satisfaction, and masticatory performance to a greater extent than RPDs by improving denture stability and enhancing support [2–20]. In particular, ISRPDs are frequently applied in patients with mandibular distal-extension edentulism, and their advantages have been shown in many studies. Hirata et al. [21–23] reported that the implant bending strain is smaller in patients with unilateral distal-extension edentulism with the use of an ISRPD when the implant’s long axis, abutment angle, and load direction are perpendicular to the occlusal plane. Matsudate et al. [24] reported that implants located in the distal area had a smaller load beneath the denture base than implants located in the mesial area in patients with unilateral distal-extension edentulism. Thus, the importance of the implant location and angle in ISRPDs has been previously demonstrated [25–34]. Conversely, with respect to RPDs, many studies [35–37] have shown that the dynamic behavior of the abutment teeth, and denture bases depend on the retainer type; in particular, higher rigidity of direct retainers reduces the dynamic behavior of the abutment teeth and denture base. However, the effect of the type of tooth-borne retainer on displacement of the abutment teeth and dentures in ISRPDs remains unclear. In particular, ISRPDs are considered to be effective prosthetic methods in patients with unilateral mandibular distal-extension edentulism, although the RPDs in these edentulous spaces are difficult to stabilize. Therefore, we performed a mechanical simulation study involving a resin mandibular model to evaluate the effect of the rigidity of a tooth-borne retainer on the dynamic behavior of the direct abutment teeth and denture base in RPDs and ISRPDs.

## Materials and methods

### Fabrication of resin mandibular model for mechanical simulation and denture from patient’s computed tomography images (Fig. 1)

A three-dimensional image in stereolithography format was constructed from computed tomography (CT). Digital Imaging and Communications in Medicine® images of a woman in her 50s who had unilateral mandibular distal-extension edentulism and was missing the first molar, second molar, first premolar, and second premolar. Mimics Innovation Suite 21.0 (Materialise, Leuven, Belgium) was used to extract the mandible and tooth shapes from the CT images. A resin mandibular model for mechanical simulation was fabricated using a three-dimensional printer (Formlabs printer; Formlabs, Inc., Somerville, MA, USA) from stereolithography data. Abutment teeth, to which a direct or indirect retainer was applied, were cut out from a polymethyl methacrylate disk (resin disk; Yamahachi Dental Mfg., Co., Aichi, Japan) using a computer-aided manufacturing system and milling machine (S-WAVE DWX-50; Shofu Inc., Kyoto, Japan). The resin-facing crown was made of gold–silver–palladium alloy and cemented to the right canine as a direct abutment tooth, and full-cast crowns were made of gold–silver palladium alloy and cemented to the left first premolar, second premolar, and first molar of indirect abutment teeth using resin adhesive cement (PANAVIA V5; Kuraray Noritake Dental Inc., Tokyo, Japan). The canine tooth crown length was about 10 mm, and the root length was about 15 mm.

**Fig. 1:**
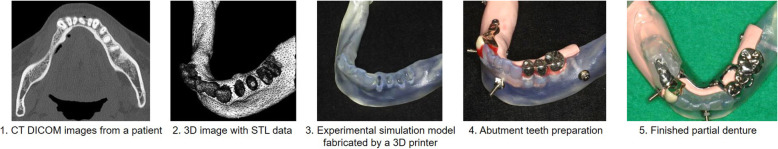
Fabrication process of the resin mandibular model for mechanical simulation and denture

An approximately 0.3-mm-thick artificial periodontal ligament made of silicone impression material (Fit Checker; GC, Tokyo, Japan) was inserted around the roots of the direct and indirect abutment teeth according to the report by Kono et al. [29]. Artificial mucosa of 2.0-mm thickness was made using silicone impression material (Correcsil; Yamahachi Dental Mfg., Co.) applied to the edentulous area of the mandibular simulation model [25, 28, 29].

**Fig. 2:**
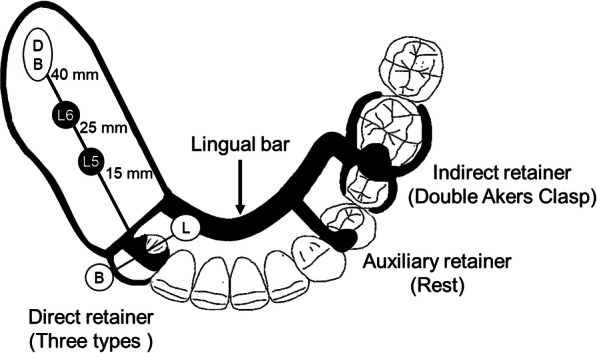
Schematic illustration of a mandibular experimental distal-extension removable partial denture

**Fig. 3:**
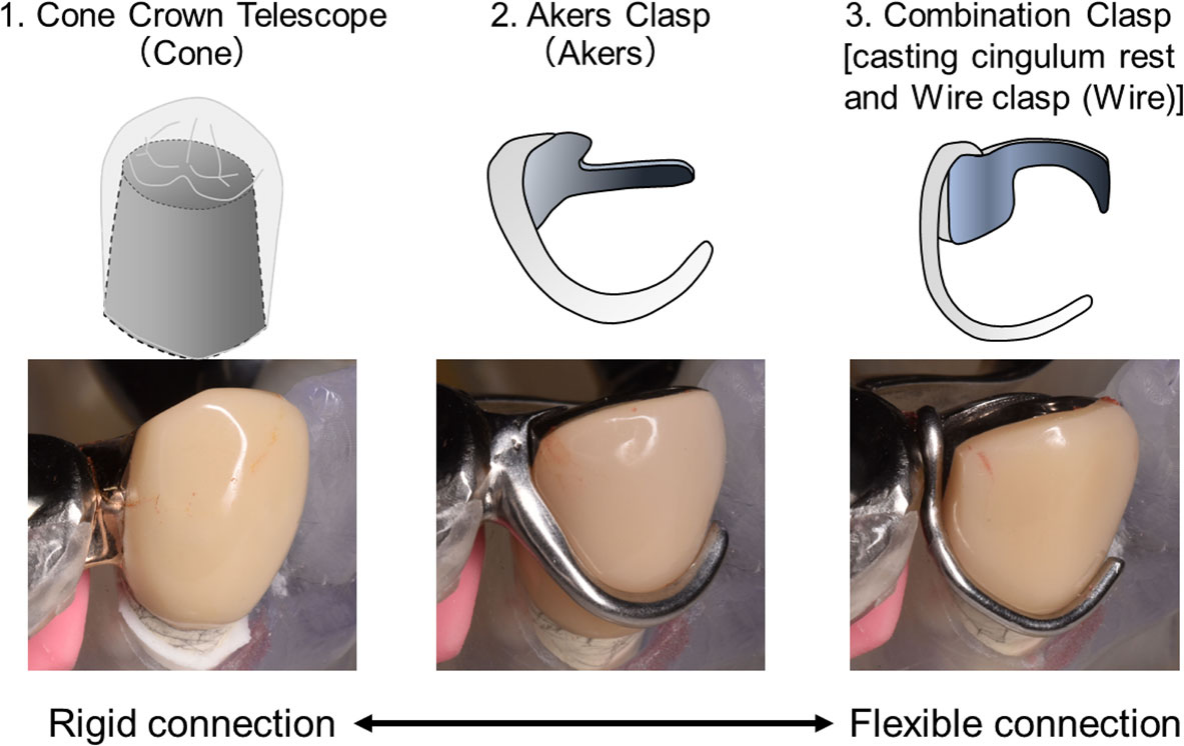
Schematic illustration of the three different types of direct retainers used to evaluate rigidity

The fabricated experimental RPD is shown in Fig. 2. The cobalt–chromium alloy cast metal framework denture consisted of a lingual bar as a major connector, a double Akers clasp at the left second premolar and first molar, and an occlusal rest at the left first premolar. Three types of direct retainers with different connecting rigidities were evaluated at the right canine: a cone crown telescope (Cone) made of gold–platinum alloy, an Akers clasp (Akers), and a wrought wire clasp with a cast cingulum rest (combination clasp, (Wire)) made of cobalt–chromium alloy (Fig. 3). Three direct retainers were manufactured for each type and fixed to the cast metal framework with two screws. The occlusal surface of the edentulous area was prepared parallel to the occlusal plane.

An implant (4.0 mm in diameter, 8.0 mm in length; IAT EXA plus 2-stage screw; Nippon Piston Ring Co. Ltd., Saitama, Japan) was inserted at the position equivalent to the first molar in the edentulous residual ridge perpendicular to the occlusal plane, and a 5.0-mm-high healing abutment was connected to the implant. The denture base of the ISRPD was relined and touched to the top of the healing abutment with acrylic resin. Consequently, the healing abutment served a role in supporting the denture.

### Preliminary examination of the artificial periodontal ligament and simulated mucosa

Artificial periodontal ligament and simulated mucosa were prepared according to previous studies [25, 28, 29], and preliminary examinations were performed to examine the validity of the study design. We measured vertical displacement in the canine tooth with an artificial periodontal ligament in this study. Vertical load ranged from 100 gf to 1000 gf. In the simulated mucosa, we applied a vertical load over surface area measuring 1.0 cm^2^ in diameter. Vertical load ranged from 1 gf/mm^2^ to 10 gf/mm^2^.

### Measurements of dynamic behavior in the ISRPD or RPD

**Fig. 4:**
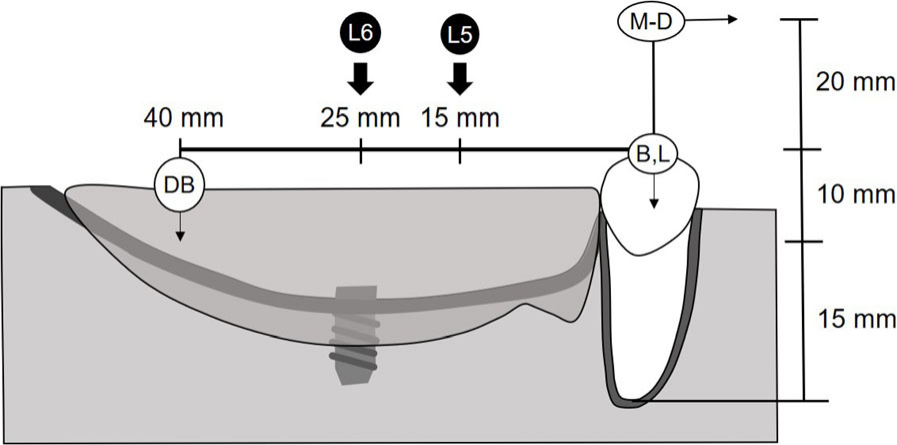
Schematic illustration of the resin mandibular model for mechanical simulation and denture

Displacement were evaluated at four measurement points (Figs. 2 and 4). DB, to measure vertical displacement, was located 40 mm distal to the center of the canine on the denture base. M–D, to measure mesial displacement, was located 20 mm upward from the top of the canine. B and L, to measure vertical displacement on the buccal and lingual sides, were located 20 mm buccally and lingually from the top of the canine teeth and perpendicular to the alveolar crest, respectively. Laser displacement sensors were used to measure displacement of the abutment teeth (LK-010; KEYENCE, Osaka, Japan, and PCD-430A; Kyowa, Tokyo, Japan).

A vertical load of 50 N was applied in the occlusal plane at points L5 and L6 (Figs. 2 and 4). L5 and L6 were located at 15 mm and 25 mm from the center of the canine on the occlusal plane, respectively.

Three direct retainers for each type were made (total of nine retainers). The dynamic behavior under a load at L5 and L6 was measured six times for each retainer.

### Statistical analysis

We created a graph of the results of the preliminary examination showing the relationship between load and displacement and compared our results with those in previous studies.

The dynamic behavior of the abutment teeth and dentures was statistically compared between ISRPDs and RPDs using the Wilcoxon rank sum test and among the three direct retainers using the Kruskal–Wallis test and Steel–Dwass test. All statistical analyses were performed using JMP® 14.0 (SAS Institute Inc., Cary, NC, USA) with a significance level of 0.05.

## Results

### Preliminary examination

**Fig. 5:**
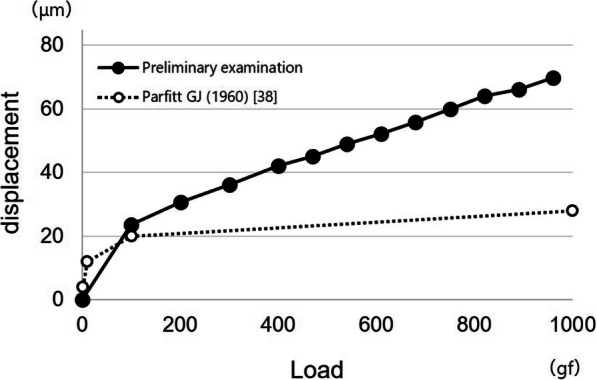
Load displacement curve for the artificial periodontal ligament. Black circles indicate data from the preliminary examination of the artificial periodontal ligament in this study; white circles indicate data from Parfitt GJ (1960) [38]

**Fig. 6:**
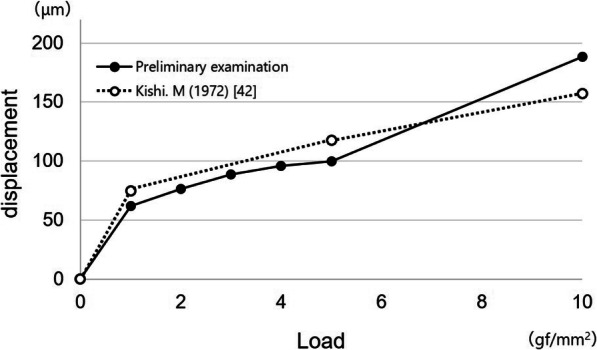
Load displacement curve for the simulated mucosa. Black circles indicate data for the preliminary examination of the simulated mucosa in this study; white circles indicate data from Kishi M (1972) [39]

The results of the preliminary examination are shown in Figs. 5 and 6. Vertical displacement of the canine tooth with the artificial periodontal ligament was 23 μm at 100 gf, 36 μm at 300 gf, and 70 μm at 1000 gf.

When pressure was applied to the simulated mucosa over a surface area of 1.0 cm^2^, vertical displacement was 62 μm at 1 gf/mm^2^, 100 μm at 5 gf/mm^2^, and 188 μm at 10 gf/mm^2^.

### Dynamic behavior of the direct abutment teeth and the denture base

**Fig. 7:**
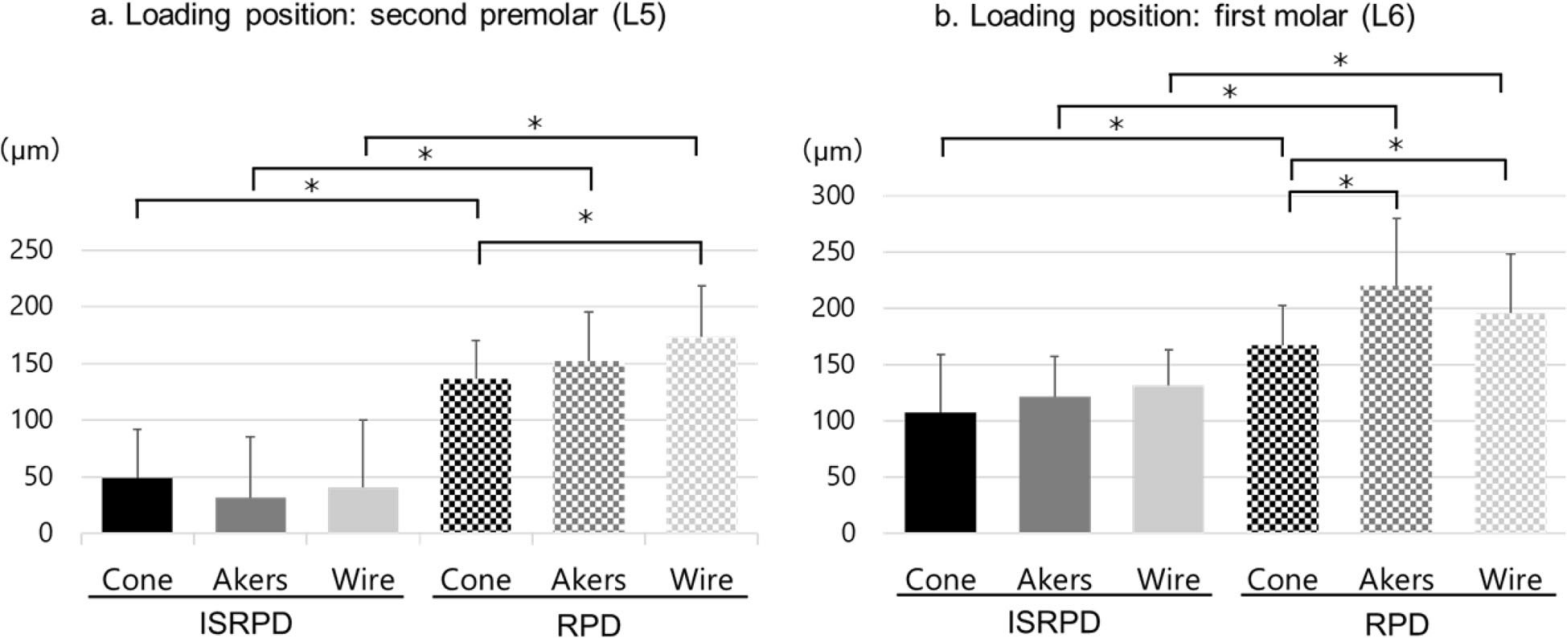
Amount of vertical displacement at the end of the denture base. Data are expressed as the mean displacement at the end of the denture base of each direct retainer, and error bars represent the standard deviation

**Fig. 8:**
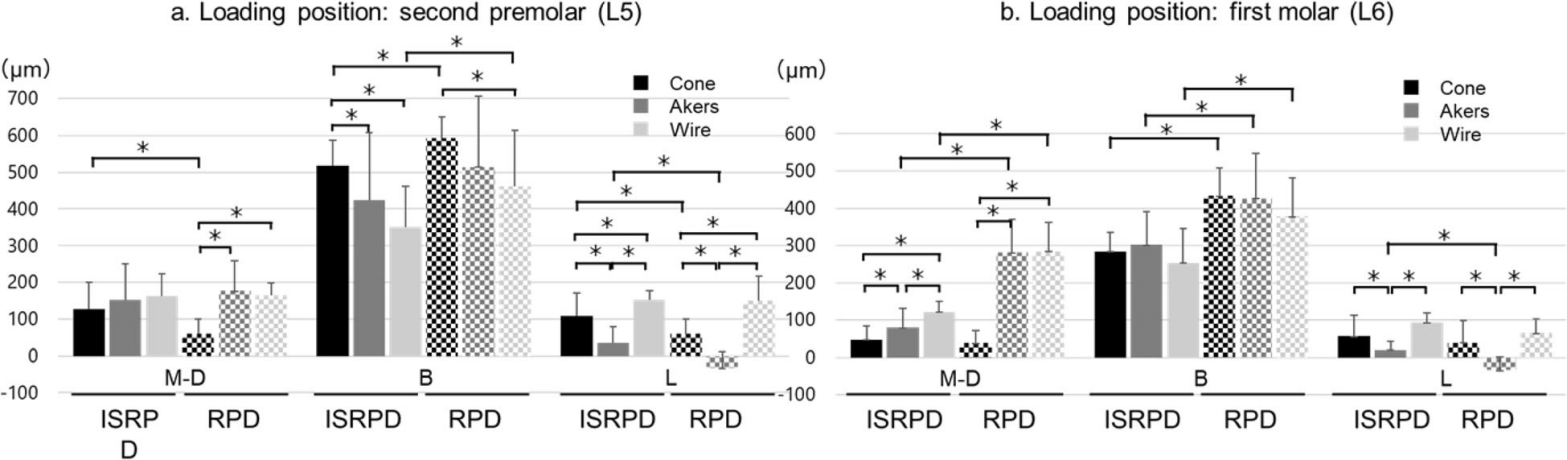
Displacement at the direct abutment teeth. Data are expressed as the mean displacement at the direct abutment teeth of each direct retainer, and error bars represent the standard deviation

The dynamic behavior of the direct abutment teeth and the denture base depended on the location of the loading point. At L5, which was located mesial to the implant, displacement of the direct abutment teeth was larger, and that of the denture base was smaller than that at L6, which was located directly above the implant (Figs. 7 and 8). Vertical displacement at L was smaller than that at B, which indicated that the canine, as the direct abutment tooth, inclined toward the buccal side (Fig. 8).

### Comparison between the dynamic behavior of the RPD and ISRPD

Displacement in the DB direction with ISRPDs was significantly smaller than that with RPDs, regardless of the rigidity of the direct retainers and loading position (*P* < 0.001 for all tests; Fig. 7). Conversely, if a load was applied to the first molar (L6), then the dynamic behavior of the direct abutment teeth with the Akers and Wire clasp in RPDs showed significantly more mesial displacement than in ISRPDs (*P* < 0.001; Fig. 8). Furthermore, mesiodistal displacement in ISRPDs with the Cone tended to show more mesial displacement than that in RPDs (*P* = 0.001 at L5 and *P* = 0.527 at L6; Fig. 8). Finally, for buccolingual displacement, the direct abutment teeth with the Cone and Wire in RPDs were displaced significantly more buccally than those in ISRPDs (Cone: *P* < 0.001 at L5 and L6, Wire: *P* = 0.019 at L5 and *P* < 0.001 at L6).

### Comparison between the three types of direct retainers with different connecting rigidities

DB displacement in the RPD was smallest with the Cone among the three direct retainers (*P* = 0.490 compared with Akers and *P* = 0.041 compared with Wire at L5, *P* = 0.005 compared with Akers and *P* = 0.002 compared with Wire at L6; Steel–Dwass test). However, in the ISRPD under loads at L5 and L6, there was no significant difference in displacement of DB among all retainers (*P* = 0.165 at L5 and *P* = 0.600 at L6; Kruskal–Wallis test; Fig. 7).

When a load was applied at L5, the abutment tooth with the Cone was displaced most buccally among all retainers in the ISRPD (*P* = 0.023 compared with Akers and *P* < 0.001 compared with Wire; Steel–Dwass test; Fig. 8). However, when a load was applied at L6, there was no significant difference in displacement on the buccal side among all retainers in the ISRPD (*P* = 0.162; Kruskal–Wallis test; Fig. 8).

## Discussion

Many basic research studies and clinical evaluations of ISRPDs have been conducted [2–20]. These studies investigated the survival rates of implants and prostheses and patient-based outcomes such as oral health-related quality of life. For example, Bassetti et al. [2] reviewed several studies on the survival rates of direct abutment teeth with ISRPDs. De Freitas et al. [3] conducted a systematic review and found that this treatment approach could represent a low-cost and beneficial rehabilitation strategy for mandibular distal-extension edentulism. Gonçalves et al. [7] reported that masticatory performance improved to a greater degree with ISRPDs than with conventional RPDs. Because the ISRPDs enhanced support by the implant at the edentulous space, the force to the abutment teeth decreased. This effect might influence the prognosis of the abutment teeth. To our knowledge, however, no study has elucidated the differences in the mechanical effect and prognosis of abutment teeth between using an ISRPD vs an RPD.

In this study, all components other than the implant and the direct retainer (e.g., major connector, denture base, and artificial teeth) were the same. Many studies have evaluated RPD retainers [35–37]; however, evidence about the denture design of ISRPDs is scarce. It was considered that the selection of direct retainers is very important because the direct retainer affects the dynamic behavior of the abutment teeth and denture base [35–37]. In this study, three types of retainers with different connecting rigidities were selected. Higher rigidity was found with the cone crown telescope, and lower rigidity was found with the Akers clasp and wrought wire clasp with cast cingulum rest. Artificial periodontal ligament and simulated mucosa were made according to previous studies [25, 28, 29]. In the preliminary examination, displacement at the vertical lower load under the conditions in this study was slightly larger, but these behaviors were similar to natural tooth and mucosa [38–42]. Therefore, the design of this experimental simulation study was considered appropriate.

Clinically, attachments, such as the ball and locators, are frequently used for ISRPDs and may have variable influence on the behavior of the dentures and abutment teeth. However, most attachments have a supportive effect for ISRPDs. In this study, we selected simple healing abutment to serve as support for implant attachment. We aimed to evaluate the effect of support by the ISRPD regardless of the type of attachments. The authors of a previous study evaluated an ISRPD mounted to a healing cap at the distal-extension edentulous space [21–23, 25, 29]. However, additional studies of the difference in the dynamic behavior of the abutment teeth and the denture with ISRPDs are needed to evaluate the characteristics of each attachment.

### Implant influence at the distal-extension residual ridge

Several basic experiments involving ISRPDs have been conducted to explore the implant position and angle and the stress in the direct abutment tooth and residual ridge. Ohkubo et al. [25] reported that the presence of an implant prevented strain on the mucosa of the residual ridge and reduced displacement of the abutment teeth in ISRPDs compared with RPDs. Moreover, the stress of the alveolar ridge was more efficiently reduced with an implant positioned perpendicular to the occlusal plane and in the posterior area. Ortiz-Puigpelat et al. [31] generated a digital model from cone-beam CT data and analyzed the behavior of ISRPDs in a patient with bilateral mandibular distal-extension edentulism. They found that placing the implant in the first molar area enhanced the biomechanical results in the peri-implant bone in ISRPDs. The results from other previous studies [25–28] also showed that insertion of an implant in the distal-extension edentulous residual ridge could reduce displacement of the direct abutment teeth and denture base. The reduction in displacement of the denture base in ISRPDs was larger under a load in the mesial area of the implant than under a load in the distal area.

In the present study, the position of the implant in the first molar region was considered to be quite adequate to suppress the dynamic behavior of the direct abutment teeth and denture base in ISRPDs compared with RPDs.

### Displacement of the abutment teeth

Displacement of the denture bases and direct abutment teeth was measured under a vertical load of 50 N in the occlusal plane. In ISRPDs, mesial displacement of the direct abutment teeth was considered to be within physiological mobility, which reportedly ranges from 25 to 100 μm [38, 43]. M-D directly reflected mesial displacement of the abutment tooth but was amplified because the measurement point was above and apart from the cusp of the canine teeth. The center of tooth rotation was two-thirds of the alveolar bone height from the root apex [44]. In the resin mandibular model for mechanical simulation used in this study, we estimated that about one-half of the obtained results reflected the true behavior of the top of the canine teeth. The buccolingual behavior of the direct abutment teeth could be estimated from the difference between the B and L values. For all measurements obtained in this study, the B values were larger than the L values. The least amount of displacement of the direct abutment teeth in the buccal direction was observed with the use of a combination clasp in ISRPDs. Additionally, the L values in this study indicated displacement in the apical direction. Regardless of the existence of an implant and the loading positions, the vertical displacement of abutment teeth with a combination clasp was larger than that with the other direct retainers. Under loading at L6, there was no difference in vertical displacement of the abutment teeth between the cone crown telescope and the combination clasp, and the vertical displacement was larger than that with the Akers clasp. This result could be explained by the mesiodistally wider cingulum rest of the combination clasp and the high rigidity of the cone crown telescope. Furthermore, the combination clasp suppressed displacement in the buccal direction because the rest was located on the lingual side. Therefore, among the three direct retainers evaluated in this study, the combination clasp would be the most suitable direct retainer for use in ISRPDs because it efficiently distributes the load along the tooth axis in the apical direction within the physiological limits. Conversely, the dynamic behavior of the denture base was dependent on the implant retainer type.

### Rigidity of the direct retainer

The rigidity of retainers affects the dynamic behavior of the abutment teeth and denture base [35–37]. Some studies have examined the stress distribution and displacement of the abutment teeth and denture base in RPDs. Saito et al. [36] suggested that rigid retainers such as a rigid precision attachment and cone crown telescope more effectively decreased displacement of the denture base than do clasps. Moreover, they indicated that a rigid connection tended to concentrate more stress at the direct abutment teeth than did a flexible connection. Igarashi et al. [35] investigated the stress distribution and abutment tooth mobility in patients with distal-extension RPDs in vivo. They reported that the denture base sharing loads of high-rigidity retainers were smaller than those of flexible connecting retainers. Additionally, Itoh et al. [37] investigated the effect of the rigidity of direct retainers on displacement of the abutment teeth and denture base. They found no significant difference in relation to the displacement of the abutment teeth in the apical direction. However, buccal displacement with a wrought wire clasp was larger than that with a cone crown telescope. The present study yielded opposing results. One potential explanation for the discrepancy might be that we measured the mandibular canine teeth as abutment teeth, whereas previous studies evaluated the maxillary first premolar. The tooth type could affect the tooth axis inclination and the support area. In addition, the wrought wire clasp caused significantly more displacement of RPDs in the apical direction than did the cone crown telescope. Igarashi et al. [35] and Itoh et al. [37] suggested that the high rigidity of direct retainers decreased displacement of the dentures. As these studies showed, rigid direct retainers decrease the dynamic behavior of dentures. However, these retainers might result in excessive loading beyond the physiological range for direct abutment teeth.

In the present study, although vertical displacement of the denture bases and direct abutment teeth was dependent on the rigidity of the direct retainers in RPDs, no significant difference was observed in ISRPDs regardless of the retainer type. However, high rigidity caused the largest displacement of abutment teeth with loading at L5, which increased the amount of load on the abutment teeth. Although a load applied distal to the implant was likely to be more strongly transmitted to the implant, a load applied mesially increased the amount of load on the abutment teeth. Therefore, it was difficult to reduce stress on the direct abutment teeth in ISRPDs, similar to RPDs.

To our knowledge, no study has examined the differences between ISRPDs and RPDs from the viewpoint of the prognosis of the remaining teeth. Ishida et al. [45] reported that differences in retainer rigidity influence the survival rates and complication-free rates of abutment teeth. They observed no significant difference in the survival rates between two types of prostheses, but double crown prostheses with high-rigidity retainers exhibited more complications than other retainer types. The prognosis of the remaining teeth in patients with partial edentulism might be improved if high rigidity is not required for retainers.

The clinical significance of the partial prosthesis design is considered to lie in suppression of movement of the prostheses and prevention of loss of the remaining tissue such as the abutment teeth and alveolar ridge. Higher rigidity of direct retainers might not be necessary from the viewpoint of a decreased load on the abutment teeth because displacement of the denture base decreases irrespective of the direct retainer type in ISRPDs. Thus, ISRPDs with a combination clasp would be appropriate to minimize denture movement and reduce the load on the remaining teeth.

In this study, the simulation model used to analyze the dynamic behavior of the denture and direct abutment teeth was generated using CT data from a single patient. Therefore, the generalizability of the results may be limited. Further analysis of dynamic behavior using data from multiple patients, stress distribution using finite element analysis, and functional analysis in actual clinical practice are required. Moreover, as only displacement was measured in this simulation study, the influence of the three kinds of tooth-borne retainers on the pressure distribution to the implant is unclear. It would be necessary to combine the analysis using strain gauges to obtain more reproducible results in the future.

Furthermore, this study demonstrated that the characteristics of the tooth-borne retainer might affect the stresses on the tooth; therefore, further clinical research should be performed. Clinically, the dynamic behavior of the abutment teeth and dentures in the partial edentulism evaluated in this study and another forms of bilateral distal-extension edentulism appear to be similar depending on the various rigidities of the retainers. Conversely, the dynamic behaviors in other forms of edentulism, such as unilateral distal-extension edentulism and intermediate edentulism, differed from our study because of the direction of the denture rotation. However, denture support by the dental implant is expected to highly suppress the behavior of the abutment teeth and the denture with ISRPDs. Therefore, analyses of various forms of edentulism and ISRPD use are required.

## Conclusions

This study showed that application of an implant in the distal-extension edentulous area significantly reduced the amount of vertical displacement at the denture base. Our results also suggested that ISRPD movement did not depend on the type of direct retainer. Conversely, our results suggested that an ISRPD with a combination clasp distributes the load along the direct abutment tooth axis in the apical direction and suppresses displacement in the buccal direction compared with other types of retainers.

## Data Availability

The datasets used or analyzed during the current study are available from the corresponding author on reasonable request.

## Abbreviations

RPD: Removable partial dentures
ISFP: Implant-supported fixed prostheses
ISRPD: Implant-supported removable partial dentures
CT: Computed tomography
